# Family endowments and multidimensional poverty among urban older adults living alone

**DOI:** 10.3389/fpubh.2025.1585456

**Published:** 2025-09-05

**Authors:** Xingxing Yin, Jinghang Cui, Ruirui Bai, Yifan Wu, Jiayue Ma, Yifei Zhai, K. Jason Crandall

**Affiliations:** ^1^School of Social Development, East China Normal University, Shanghai, China; ^2^Center for Applied Science in Health and Aging, Western Kentucky University, Bowling Green, KY, United States; ^3^Department of Economics, University of Wisconsin-Madison, Madison, WI, United States; ^4^School of Psychology and Cognitive Science, Beijing Language and Culture University, Beijing, China; ^5^Sports Science Institution, Nanjing University, Nanjing, China

**Keywords:** urban older adults living alone, multidimensional poverty, family endowments, household older adults care capacity, intergenerational support

## Abstract

**Background:**

Disparities in family endowments play a pivotal role in shaping the multidimensional poverty among urban older adults living alone (UOALA).

**Methods:**

This study conducted an empirical analysis using data from 1,656 UOALA in five different cities in China to investigate the relationship between family endowments and the occurrence of multidimensional poverty among UOALA.

**Results:**

The findings illustrate an expanding and deepening scale of poverty among UOALA, particularly manifested in dimensions of physical health and mental well-being. Females, super-older adults, and those residing in non-first-tier cities exhibit higher levels of poverty incidence and depth. The study also reveals that while the number of children in a family does not significantly impact multidimensional poverty in UOALA, factors such as children’s proximity of residence and financial situation play a significant role. Moreover, gender differences are observed in the influence of sons and daughters on the poverty status of UOALA.

**Conclusion:**

Enhancing family older adults care support capacity and improving societal welfare levels emerge as critical policy measures to prevent UOALA from falling into Multidimensional Poverty.

## 1 Introduction

As the global population ages at an accelerating pace, ensuring that older adults have equal access to the benefits of development has become the focal point of older adults care in China’s new era. The extension of life expectancy and changes in social roles during old age make older adults more vulnerable to risks such as illness, disability, and accidents that can lead to poverty. The resulting costs of care, medical treatment, and support will be a heavy burden on both families and society. The emergence of poverty in old age not only decreases the well-being of older adults but also has a negative effect on the overall sense of achievement within society and even on the healthy and sustainable development of the national economy. Against the backdrop of modernizing families, some scholars predict that by the 2030s and 2040s, 60–70% of married children in urban areas of China will no longer live with their parents (1), leading to a significant increase in the proportion of older adults living alone. This situation will transfer some of the risks associated with older adults care in families to the public domain, creating macro-level societal risks.

Early research on older adult poverty in China largely focused on rural areas. With socioeconomic development, urban income gaps have widened and prices have risen, yet social security coverage remains limited, further exacerbating urban poverty. Azeem et al. (2) used a regional comparative study to find that urban households are more vulnerable than rural ones and, when faced with specific shocks, are more likely to fall into poverty. In recent years, the number of urban older adults in poverty has increased significantly; however, because the definition of urban poverty is ambiguous, their plight has been overlooked (3). Against the backdrop of population aging and changes in household structure, the marginalization and impoverishment of urban older adults living alone have become especially pronounced. Compared with traditional income-expenditure poverty analysis, studying multidimensional poverty is significant because it can identify and measure the complex factors that drive people into poverty. Older adults living alone share common poverty-inducing characteristics with the broader older adult population but also face unique risks; yet, because their issues are difficult to identify and measure, they become a blind spot in national poverty alleviation efforts. Therefore, measuring the poverty status of urban older adults living alone from a multidimensional perspective, exploring the factors that drive their impoverishment, and discussing how to achieve poverty alleviation for this special older adult group under the “aging before getting rich” socioeconomic conditions have become important topics of concern for both academia and government.

Research shows that household structural characteristics are closely related to older adult poverty. Existing studies examine this relationship from material, human, and social capital perspectives, but their conclusions differ. Some researchers argue that households have a positive effect on alleviating older adult poverty: Wang and Zhou (4) found that intergenerational support is the main mode of economic support in Chinese older adult households—when there are more adult children, there is a greater likelihood of forming economic support relationships; Hu (5) maintained that, under an authoritative filial piety structure, children provide stronger economic support to their older parents. Other studies indicate that household endowment does not necessarily alleviate older adult poverty. In the context of demographic transition, the effect of household endowment on older parents falling into poverty is significant but inconclusive (6). Song et al. (7) found that older adults with male offspring have a higher likelihood of falling into economic poverty, and Shi (8) argued that social competition and the cost of establishing a family force the older generation to work harder to sustain family regeneration in old age, making them prone to an “intergenerational transmission of poverty”.

Against this backdrop, this paper uses the “Survey Data on the Status and Needs of Urban Older Adults Living Alone,” collected by the research team of the National Social Science Foundation’s major project “Research on the Home-Based Older Adult Care Security System for China’s Urban older adults Population in the Next Decade,” to conduct an empirical study of poverty among older adults living alone from a household dimension. This study aims to advance in the following respects: First, whereas existing research is mostly based on all age groups, this paper selects the special older adult group living alone as its study population, thus filling a gap in the literature on poverty among special groups. Second, unlike prior studies that focus on a single economic indicator, this paper introduces the important concept of “multidimensional poverty” to depict the extent to which older adults living alone fall into poverty across economic, physical, functional ability, social participation, and psychological dimensions, as well as to reveal heterogeneity within this population. Third, this study delves into the concrete household life practices of older adults living alone to explore the household structural factors underlying their multidimensional poverty. By doing so, it both expands the understanding of older adult poverty—providing a reference for setting poverty standards for special groups—and, to some extent, compensates for the existing lack of research on the vulnerable subgroup of older adults living alone from a household perspective, offering sustainable insights for rethinking the challenges of older adult poverty governance.

### 1.1 Research method

#### 1.1.1 Multidimensional poverty measurement

This paper adopts the dual-cutoff poverty identification and measurement method proposed by Oxford scholars Alkire and Foster (9), referred to as the AF method, to construct a multidimensional poverty index—that is, by using deprivation cutoffs for each dimension and a poverty cutoff across all dimensions to define multidimensional poverty. An n*d matrix [x₍ᵢⱼ₎] represents the status values of *n* individuals across d dimensions. Any element x₍ᵢⱼ₎ in X denotes the poverty identification value for individual i(i = 1, 2, …, n) in dimension j(j = 1, 2, …, d). The element zj(zj > 0) denotes the deprivation cutoff for dimension, given the matrix xij, ne obtains the corresponding deprivation matrix g0 = [ $g_{\mathrm{ij}}^{0}$ ], where g^0^₍ᵢⱼ₎ = 1 if x₍ᵢⱼ₎ < zⱼ, and g^0^₍ᵢⱼ₎ = 0 if x₍ᵢⱼ₎ ≥ zⱼ—indicating that if individual i’s value in dimension j falls below the deprivation cutoff, it is assigned 1; otherwise, it is assigned 0.

If an individual is deprived in multiple dimensions, a multidimensional poverty cutoff k is introduced. For individual i, their weighted deprivation score is $c_{i}=\sum_{j=1}^{d}w_{j}g_{\mathrm{ij}}^{0}$ , where wⱼ is the weight for indicator j, and cᵢ ranges from 0 to 1. I(k) is the poverty identification function, when $c_{i}\geq I(k)$ , the individual is classified as multidimensionally poor. Summing all classified multidimensionally poor individuals yields (q), and the multidimensional poverty headcount ratio (H) is q/n, where *n* is the total sample size.

The average deprivation share (A), which represents poverty intensity, is the proportion of deprived dimensions (out of the total number of dimensions) averaged across all multidimensionally poor individuals:


$$A=\sum_{i=1}^{q}c_{i}(k)/q=\sum_{i=1}^{q}\sum_{j=1}^{d}w_{i}g_{\mathrm{ij}}^{0}(k)/q$$

Based on the AF method, the multidimensional poverty index M = H * A can be further expressed as


$$M_{0}=\frac{1}{n}\sum_{i=1}^{n}\sum_{j=1}^{d}w_{j}g_{\mathrm{ij}}^{0}(z_{\mathrm{ij}})I(k)$$

If one wishes to further understand deprivation across different dimensions, regions, age groups, or genders, one can perform dimensional decomposition and subgroup decomposition of the multidimensional poverty index, and calculate each subgroup’s contribution rate to overall multidimensional poverty. The poverty index for dimension j can be expressed as:


$$M_{j}=\frac{1}{n}\sum_{i=1}^{n}w_{j}g_{\mathrm{ij}}^{0}(z_{j})I(k)$$

The contribution rate of each dimension to the total poverty index is P = M_j_/M_0_, where the sum of all dimension contribution rates is 1. In addition to dimension decomposition, sub-group decomposition can also be conducted. If there are S sub-groups (s = 1, 2, …, S), where $\theta_{s}$ is the proportion of sub-groups in the total population n, and $M_{s}$ represents the multidimensional poverty index of each sub-group, with the total multidimensional poverty index $M_{0}=\sum_{s=1}^{S}\theta_{s}M_{s}$ , meaning that the multidimensional poverty index of each sub-group is weighted by population.

#### 1.1.2 The logit model for exploring the correlation between family endowments and multidimensional poverty among UOALA

In order to empirically examine the correlation between family endowments and multidimensional poverty among UOALA, the status of multidimensional poverty among UOALA (pover) is set as the dependent variable, with a value of 1 assigned to those in multidimensional poverty and a value of 0 assigned to those not in multidimensional poverty, with family endowments as the core explanatory variable. The following logit model is constructed for empirical analysis:


$$\ln\frac{P({\text{pover}}_{i}=1)}{1-P({\text{pover}}_{i}=1)}=\beta_{0}+\beta_{1}{\mathrm{fam}}_{i}+{\mathrm{\gamma Z}}_{i}+\varepsilon$$

where P represents the probability of multidimensional poverty among UOALA (pover = 1); m is the logarithm of the odds ratio of multidimensional poverty among UOALA, also known as the logit value of multidimensional poverty among UOALA; fam stands for family endowments; Z represents control variables; and *ε* denotes the random disturbance term.

## 2 Analysis framework and research methods

### 2.1 Analysis framework

Due to the limitations of development stages and research perspectives, in the past studies on poverty and anti-poverty practices, the research focus was usually based on material aspects of explicit poverty elements, while lacking consideration of relevant non-material factors leading to poverty. In fact, in the traditional concept of poverty in China, poverty has multi-dimensional attributes. Feng et al. (10) discovered that the Chinese term “pín kùn” (poverty) is comprised of two concepts, where “pín” signifies scarcity and “kùn” represents being constrained, i.e., individual material deprivation and limited development capabilities. Sen (11) proposed the theory of multidimensional poverty, which suggests that poverty is not just related to low income, but also about lacking the ability to create income and opportunities. He further pointed out that low monetary income levels cannot fully explain the causes of poverty, as poverty constraints originate from multiple dimensions including illness, education, housing, public facilities, reflecting a lack of a variety of “capabilities.” Poverty measurement is not limited to a single dimension such as income or consumption standards, as multidimensional poverty has received widespread attention and application due to its better targeting effectiveness (12). Previous studies on poverty in old age have been limited in scope and have failed to adequately consider the various forms of poverty in old age and the complex relationships among different factors. This has led to a lack of scientific rigor in establishing poverty thresholds. The Multidimensional Poverty Measure addresses the limitations of solely measuring income or expenditure, instead examining the deprivation situations of UOALA through multiple functional perspectives.

From the perspective of multidimensional poverty measurement indicators, current research often utilizes methods such as the Alkire-Foster method, the Human Poverty Index, the Multidimensional Poverty Index, etc. (13). Among these, the Alkire-Foster method, building on the counting approach and integrating Sen’s “capability set” theory, constructs a measurement system for multidimensional poverty and uses dual cutoffs to define poverty based on overall and dimensional deprivation thresholds (9). Because of its robust functionality and capacity to capture the range, depth, and severity of poverty from various viewpoints, it has emerged as one of the most extensively utilized approaches for assessing multidimensional poverty. This paper argues that while financial situation is indeed a core indicator for measuring poverty, non-monetary dimensions such as physical health, functional capacity, social participation, and mental health are also key indicators for assessing poverty status. These five dimensions interact to some extent, collectively forming the development goals and means of the later life of UOALA.

## 3 Data sources and variable definitions

### 3.1 Data sources

This study utilizes data from “Research on the Home-Based Care System for the Urban Older Adults in China in the next ten years,” a project granted by the National Social Science Fund of China. From November 2013 to May 2015, a stratified multistage sampling method was employed in five cities: Shanghai, Guangzhou, Chengdu, Hohhot, and Dalian. UOALA older adults 70 and above who had resided in the local area for at least six months (by “living alone,” we mean “sleeping alone at night”) were selected according to the “administrative district, street, neighborhood and household” they belong to in the five cities. The survey questionnaires included a preliminary questionnaire, a long questionnaire and a short questionnaire. The preliminary questionnaire was used to assess cognitive functioning in the subjects on a scale of 1 to 10, with higher scores indicating better cognitive test performance. If the total score was 6 or above, the long questionnaire was assigned to the subject. If the total score was 5 or below, the short questionnaire was used. This study utilized the data from the long questionnaire of the survey, which underwent data cleaning procedures to remove samples with missing values or those without children, resulting in a final sample of 1,656 UOALA.

### 3.2 Definition of variables

#### 3.2.1 Measurement of multidimensional poverty

Establishing a multidimensional poverty index system for UOALA requires full consideration of the realities of economic and social development, as well as a certain degree of forward-thinking to align with future development goals. Current research on multidimensional poverty lacks uniformity in indicator selection due to differences in understanding and data limitations. To address this issue, this paper proposes measuring 15 indicators across 5 dimensions (financial situation, physical health, functional capacity, social participation, and mental health) based on the United Nations Development Program Multidimensional Poverty Index and China’s government practices in poverty alleviation (see Table 1). This paper follows the common practice in domestic and international studies by assigning equal weights to each dimension (9, 14–16), with indicators within each dimension also equally weighted. Related research has employed artificial neural networks (ANN), principal component analysis, and other methods to calculate indicator weights and compared those results with equal-weighting for robustness checks, finding minimal differences (17–19), thus validating the reliability of the equal-weighting approach.

**Table 1:** Definitions of poverty dimensions, indicators, and deprivation thresholds.

Dimensions	Norm	Deprivation threshold	Weights
Financial situation (1/5)	Personal income	Assign a value of 1 if the monthly average income is less than 2000 RMB.	1/20
	Deficit	Assign a value of 1 if the expenditure range is higher than the income range.	1/20
	Financial self-assessment	Assign a value of 1 if one feels their financial situation is difficult or very difficult.	1/20
	Social security	Assign a value of 1 if one receives social assistance (including subsistence allowances, etc.) or does not receive any older adults care benefits.	1/20
Physical health (1/5)	Health self-assessment	Assign a value of 1 if one feels their health condition is not very good or not good.	1/15
	Major illnesses	Assign a value of 1 if one has the following diseases: hypertension, coronary heart disease, stroke, diabetes, chronic bronchitis in old age, cancer, rheumatism, chronic kidney disease, osteoarthritis, depression, senile dementia, Parkinson’s disease, or others.	1/15
	Health expectancy	Assign a value of 1 if one is very worried or somewhat worried about their physical condition.	1/15
Functional capacity (1/5)	Self-care capability	Assign a value of 1 if one is partially or completely unable to take care of themselves.	1/15
	Daily life	Assign a value of 1 if the total score of tasks such as eating, dressing, getting in and out of bed, moving indoors, washing face and brushing teeth, using the toilet, bathing, climbing stairs, urinary incontinence, and fecal incontinence is below 60, with a scale of 10 points for no difficulty in the above items, 5 points for some difficulty, and 0 points for very difficult.	1/15
	Routine activities	Assign a value of 1 if the total score of tasks such as shopping, going out, cooking, doing household chores, washing clothes, using the phone, taking medication, and managing finances is below 48.	1/15
Mental health (1/5)	Feelings of loneliness	Assign a value of 1 if one often feels lonely or sometimes feels lonely.	1/15
	Self-efficacy	Assign a value of 1 if one is worried or anxious that they may not be able to do things that people expect of them.	1/15
	Life purpose	Assign a value of 1 if one sometimes or frequently feels that life is purposeless.	1/15
Social participation (1/5)	Social activities	Assign a value of 1 if one has not participated in any of the following: community or resident activity center, senior activity room or center, community cultural center or cultural room, various senior schools, outdoor or indoor fitness activities.	1/10
	Participation concerns	Assign a value of 1 if one has difficulties in participating in five out of the following activities: entertainment activities, sports and fitness activities, learning in senior schools (including remote education for seniors), volunteering, participating in religious or other spiritual activities.	1/10

##### 3.2.1.1 Financial situation

Rank and Hirschl (20) found that in old age, due to a lack of direct economic production activities, the stability of income level and income structure decreases, and the incidence of poverty is higher. Poverty is not only manifested in low income or consumption levels, but also in the lack of subjective satisfaction (21). Therefore, this study chooses “personal income,” “deficit,” “financial self-assessment,” and “social security” as indicators of poverty measurement in the dimension of financial situation.

##### 3.2.1.2 Physical heath

“Health poverty” is a core concept in the international public health policy system, often used to describe the state of older adults as being disabled by illness (22). This paper draws on “Long-term Multidimensional Health Poverty Index (23) to comprehensively assess the physical health status of UOALA from perspectives such as health self-assessment, major illnesses, and health expectancy.

##### 3.2.1.3 Functional capacity

The functional capacity of UOALA is also one dimension measured in assessing poverty. Ding (24) found that the lack of happiness in disabled older adults is due to the loss of functional capacity, which hinders their development of material economic abilities. In this sense, the measurement of functional capacity plays a positive role in preventing the problem of poverty in old age. Therefore, the study selected common indicators for older adults such as “self-care capability,” “daily life,” and “routine activities” to reflect the daily functional capacity of UOALA.

##### 3.2.1.4 Mental health

The mental state of UOALA is influenced by both individual and environmental factors. In old age, mental poverty mainly manifests in emotional experiences such as happiness, satisfaction, and feelings of loneliness. Therefore, in measuring mental health dimensions, this article uses common psychological and emotional states in old age, such as feelings of loneliness, self-efficacy, and life purpose.

##### 3.2.1.5 Social participation

In the older adults stage, not only is there a tendency to be in a state of “disconnection” in terms of economic development (25), but the transition of individual social roles in later life determines that older adults are more likely to be at risk of “social exclusion.” The vulnerability of UOALA continues to increase, making them more vulnerable to social capital poverty. This study selects “social activities” and “participation concerns” to characterize the measurement indicators of this dimension.

#### 3.2.2 Family endowments

“Family endowment” refers to all resources within the family—both innate and acquired human capital, economic capital, social capital, etc.—that exert a significant influence on the survival and development of family members (26). Current research yields no consensus on the role that family endowment plays in the late-life security of older adults parents (27), and due to intergenerational relationships, support relationships differ under different family structures (28). It should be noted that existing studies often interpret the concept of “family” based on household registration or the co-resident population structure at the time of survey. However, in China, the connotation of “family” is evidently much more complex. Shi (8) points out that the meaning of “family” in China transcends the concept of a registered household and needs to be extended along temporal and spatial dimensions. Tang et al. (29) also distinguish between “subjective family” and “objective family,” where subjective family is defined by self-identification, while objective family is distinguished by indicators such as co-residence and economic integration to demarcate a household community versus an economic community. During the actual questionnaire survey for this study, the interviewed older adults also more readily identified their kinship lineage, tending to consider all adult children—regardless of residence—as family members. In light of this, the present study attempts to transcend the concept of a registered household and deepen the understanding of poverty among older adults living alone from the dimension of the internalized, subjective family, focusing on the fundamental axis of family between solitary parents and their adult children to explore multidimensional poverty. Differences in household resource endowment among older adults living alone are reflected not only in children’s economic status but also in various non-economic elements. Because different levels of family endowment affect poverty patterns among older adults living alone in distinct ways, this paper analyzes the impact of family endowment on their multidimensional poverty from the dimensions of number of children, residential arrangements, and children’s economic status.

Hence, the explanatory variables of “family endowments” in this study encompass the number of children (sum_child); the minimum distance between UOALA and their closest children’s residence, established through questionnaire inquiries concerning the proximity of the older adults’ dwelling to that of their nearest children, as well as the minimum residence distance between them and their sons (dis_son) and daughters (dis_dau); children’s financial situation (inc), identified through questionnaire inquiries on the financial situation of children, selecting the optimal financial situation among the older adults’ children, as well as the optimal financial situation of sons (inc_son) and daughters (inc_dau).

#### 3.2.3 Controlled variable

Moreover, inspired by the research by Luo et al. (30), Zhong et al. (31), and Guo et al. (16), this study incorporates demographic variables as control factors, including gender (male, “1 for male, 0 for female”), age (self-reported by respondents), household registration (hukou, “1 for non-rural residency, 0 for rural residency”), migration status (imm, “1 for migrant, 0 for non-migrant”), level of education (edu, 1 for illiteracy or no formal schooling, 2 for primary education, 3 for lower secondary education, 4 for upper secondary education, 5 for post-secondary non-tertiary education, 6 for tertiary education), and marital status (mar, “1 for married, 2 for other”). Specifically, migration status was determined by comparing the current residence of the UOALA to their birthplace. If the birthplace and current residence matched, it was considered that the UOALA had not migrated; if they did not match, it was deemed that the UOALA had migrated (see Table 2).

**Table 2:** Descriptive statistical analysis of main variables.

Variants	Options	Sample Size	Average Value	Standard Deviation
pover	k = 0.33, multidimensional poverty = 1, non-multidimensional poverty = 0	1,656	0.51	0.50
sum_child	Number of children	1,656	2.79	1.21
sum_son	Number of sons	1,656	1.42	0.99
sum_dau	Number of daughters	1,656	1.38	1.06
dis	Minimum distance to the residence of children (Same neighborhood = 1, Different neighborhood in the same street = 2, Different streets or towns in the same city (district or county) = 3, Different cities (districts, counties) in the same province (autonomous region, municipality) = 4, Different provinces (autonomous regions, municipalities) in mainland China = 5, Outside mainland China = 6)	1,644	2.49	0.99
dis_son	Minimum distance to the residence of sons (assigned as above)	1,372	2.68	1.00
dis_dau	Minimum distance to the residence of daughters (assigned as above)	1,307	2.79	1.02
inc	Highest income of children (Very high = 1, High = 2, Medium = 3, Low = 4, Very low = 5, No income = 6)	1,640	2.70	0.78
inc_son	Highest income of sons (assigned as above)	912	2.69	0.77
inc_dau	Highest income of daughters (assigned as above)	724	2.70	0.78
male	Male = 1, Female = 0	1,656	0.30	0.46
age	Self-reported by the respondent	1,656	78.46	5.34
hukou	Non-rural household registration = 1, rural household registration = 0	1,648	0.94	0.23
imm	Migration = 1, Non-migration = 0	1,639	0.55	0.50
edu	Illiteracy or no formal schooling = 1; Primary education = 2; Lower secondary education = 3; Upper secondary education = 4; Post-secondary non-tertiary education = 5; Tertiary education = 6	1,639	2.60	1.30
mar	Married = 1, Other = 2	1,640	0.16	0.36

## 4 Empirical analysis

### 4.1 Measurement and identification of multidimensional poverty

#### 4.1.1 Measurement of unidimensional poverty

The results of unidimensional poverty incidence show that out of all 1,656 UOALA, only 28 individuals are not experiencing poverty across the 15 indicators. The rest of the UOALA, to varying degrees, fall into poverty, with the most prominent poverty incidence observed in the indicator of major illnesses, where the incidence of poverty is 88.83%. UOALA exhibit a higher level of poverty in the mental health dimension, with poverty affecting over 50% or close to 50% of individuals in three indicators: feelings of loneliness (58.15%), self-efficacy (51.39%), and life purpose (49.46%). In terms of functional capacity, the performance is the most favorable, with the poverty incidence less than 20% in three indicators: self-care capability (17.93%), daily life (2.29%), and routine activities (13.04%), see Table 3.

**Table 3:** The percentage of UOALA experiencing unidimensional poverty.

Dimensions	Indexes	Total	Male	Female	First-tier city	Non-first-tier city
Financial situation	Personal income	36.35	27.20	40.19	6.41	57.53
	Deficit	5.62	4.09	6.26	3.21	7.32
	Financial self-assessment	15.70	10.02	18.08	11.22	18.87
	Social security	5.37	2.86	6.43	2.19	7.63
Physical health	Health self-assessment	26.99	19.02	30.33	24.64	28.66
	Major illnesses	88.83	85.69	90.15	87.17	90.00
	Health expectancy	40.52	35.17	42.76	38.05	42.27
Functional capacity	Self-care capability	17.93	19.02	17.48	15.60	19.59
	Daily life	2.29	2.86	2.06	2.04	2.47
	Routine activities	13.04	13.91	12.68	11.52	14.12
Mental health	Feelings of loneliness	58.15	52.97	60.33	52.77	61.96
	Self-efficacy	51.39	47.44	53.04	55.10	48.76
	Life purpose	49.46	44.79	51.41	45.48	52.27
Social participation	Social activities	51.63	48.67	52.87	51.90	51.44
	Participation concerns	31.82	33.74	31.02	24.34	37.11

From a gender perspective, female UOALA exhibit a higher overall level of poverty compared to males, with only in the functional capacity dimension’s three indicators (self-care capability, daily life, routine activities) and the participation concerns indicator showing a lower incidence of poverty than males. When examining different cities, UOALA in first-tier cities generally experience lower levels of poverty compared to non-first-tier cities. Apart from the self-efficacy indicator where the incidence of poverty is higher in first-tier cities than non-first-tier cities, the remaining 14 indicators show that first-tier cities have lower poverty levels than non-first-tier cities. The most notable disparity is in the personal income indicator, where the incidence of poverty in first-tier cities is 6.41%, which is 51.12 percentage points lower than non-first-tier cities (57.53%).

#### 4.1.2 Measurement of multidimensional poverty

The selection of the multidimensional poverty threshold k is closely correlated with the measurement of multidimensional poverty. This study evaluates the multidimensional poverty among UOALA by varying the value of k, as illustrated in Table 4.

**Table 4:** The findings of the measurement on multidimensional poverty among UOALA.

k	Multidimensional poverty incidence (H)	Average deprivation share (A)	Multidimensional poverty index (M)
0.1	0.932	0.369	0.344
0.2	0.789	0.410	0.323
0.3	0.535	0.510	0.273
0.4	0.387	0.536	0.207
0.5	0.217	0.607	0.132
0.6	0.102	0.688	0.070
0.7	0.040	0.777	0.031
0.8	0.014	0.849	0.012
0.9	0.002	0.925	0.002
1.0	0.000	0.000	0.000

As the multidimensional poverty threshold (k) increases, there is a trend of a decrease in the incidence of poverty (H) among UOALA, an increase in the average deprivation share (A), and a decrease in the multidimensional poverty index (M). An increase in k means that the threshold for identifying poverty is rising, resulting in a reduction in the number of people meeting the criteria for multidimensional poverty and a decrease in the incidence of poverty. The average deprivation share A reflects the depth of poverty among the population, where identified UOALA are experiencing deprivation in more dimensions, increasing the depth. The majority of UOALA (78.9%) experience deprivation in at least one dimension (k = 0.2), with an intensity of deprivation of 0.410 and a multidimensional poverty index of 0.323. Ten percent (10.2%) of older adults experience deprivation in three or more dimensions (k = 0.6), with an intensity of deprivation of 0.688 and a multidimensional poverty index of 0.070. Even close to 1% of older adults experience multidimensional poverty in four or more dimensions. Overall, the incidence of poverty and intensity of deprivation in multidimensional poverty among UOALA are both high.

In order to thoroughly examine the correlation between various indicators and the degree of multidimensional poverty among UOALA, we have measured the poverty indices and contribution rates for each indicator. The specific details are shown in Table 5. The health of UOALA is noteworthy, whether it be physical health or mental health. The poverty indices for the mental health dimension and physical health dimension are relatively high, with the highest contribution rates to multidimensional poverty, followed by social participation, and lower contributions from financial situation and functional capacity. For example, with k = 0.3, the poverty indices for mental health and physical health are 0.088 and 0.076 respectively, contributing 32.28 and 27.94% to multidimensional poverty, with a combined contribution rate of 60%. Social participation, financial situation, and functional capacity have poverty indices of 0.063, 0.025, and 0.021, contributing 40% in total. The multidimensional poverty index and the contribution rates vary depending on the value of k. With increasing values of k, the differences in contribution rates to poverty among the five dimensions diminish. The contribution rates for financial situation and daily life increase continuously, while those for physical health, mental health, and social participation decrease steadily.

**Table 5:** The breakdown of the multidimensional poverty index of UOALA under various values of k, along with the contribution rates for each dimension.

k
	M0	Poverty index by dimension					Contribution rate by dimension (%)				
Financial situation		Physical health	Functional capacity	Mental health	Social participation	Financial Situation	Physical health	Functional capacity	Mental health	Social participation
0.1	0.3441	0.0312	0.1015	0.0222	0.1058	0.0835	9.07	29.49	6.45	30.73	24.25
0.2	0.3231	0.0293	0.0934	0.0219	0.1027	0.0757	9.07	28.91	6.79	31.79	23.44
0.3	0.2729	0.0246	0.0762	0.0213	0.0881	0.0626	9.01	27.94	7.82	32.28	22.95
0.4	0.2074	0.0194	0.0566	0.0192	0.0666	0.0457	9.33	27.29	9.24	32.10	22.04
0.5	0.1317	0.0127	0.0353	0.0154	0.0398	0.0284	9.65	26.84	11.68	30.23	21.60
0.6	0.0702	0.0076	0.0182	0.0101	0.0193	0.0150	10.83	25.85	14.39	27.52	21.41
0.7	0.0314	0.0037	0.0075	0.0056	0.0078	0.0068	11.82	23.95	17.68	24.85	21.71
0.8	0.0118	0.0014	0.0027	0.0024	0.0027	0.0025	12.29	22.87	20.14	23.21	21.50
0.9	0.0022	0.0003	0.0005	0.0005	0.0005	0.0005	13.51	21.62	21.62	21.62	21.62
1.0	0.0000	0.0000	0.0000	0.0000	0.0000	0.0000	–	–	–	–	–

#### 4.1.3 The measurement of multidimensional poverty across different samples

From a gender perspective, female UOALA experience more severe multidimensional poverty than male UOALA. The incidence and index of multidimensional poverty among female UOALA are consistently higher than those among male UOALA under any given value of k, as shown in Table 6. For example, at k = 0.3, the poverty incidence among male UOALA is 51.3%, which is 9.6 percentage points lower than that among female UOALA (60.9%). The multidimensional poverty index for male UOALA and female UOALA is 0.216 and 0.267, respectively, indicating a difference of 0.051 in favor of female UOALA. As the value of k increases, both the poverty incidence and index for male and female UOALA exhibit a decreasing trend, leading to an initial widening and subsequent narrowing of the gap in multidimensional poverty between the genders. The contribution rates to multidimensional poverty remain relatively stable for UOALA of different genders, with the contribution rate of male UOALA remaining below 30%.

**Table 6:** The gender breakdown of the multidimensional poverty index under different values of k, along with their respective contribution rates.

k	Multidimensional poverty incidence		Multidimensional poverty index		Contribution rate	
	Male	Female	Male	Female	Male	Female
0.1	0.906	0.943	0.314	0.357	0.269	0.731
0.2	0.730	0.813	0.288	0.338	0.263	0.737
0.3	0.513	0.609	0.236	0.288	0.255	0.744
0.4	0.333	0.410	0.176	0.221	0.251	0.751
0.5	0.168	0.237	0.102	0.144	0.229	0.771
0.6	0.086	0.109	0.058	0.075	0.244	0.753
0.7	0.029	0.045	0.022	0.035	0.207	0.785
0.8	0.008	0.016	0.007	0.014	0.175	0.836
0.9	0.002	0.003	0.002	0.002	0.264	0.631
1.0	0.000	0.000	0.000	0.000	-	-

When comparing different levels of cities, the multidimensional poverty status of UOALA in first-tier cities (including Shanghai and Guangzhou) is lower than in non-first-tier cities (including Chengdu, Dalian, and Hohhot), as demonstrated in Table 7. The incidence and index of multidimensional poverty among UOALA in non-first-tier cities are both higher than in first-tier cities at any given k value. When k = 0.3, the poverty incidence among UOALA in first-tier cities is 50.6%, which is 12.8 percentage points lower than in non-first-tier cities (63.4%). The multidimensional poverty index for UOALA in first-tier cities and non-first-tier cities is 0.225 and 0.306, respectively, with non-first-tier cities being 0.081 higher than first-tier cities. As k values increase, the incidence and index of multidimensional poverty among UOALA in both first-tier and non-first-tier cities decrease, with regional differences in multidimensional poverty first widening and then narrowing. The contribution rate of first-tier cities to poverty decreases with increasing k values, while the contribution rate of non-first-tier cities to poverty gradually rises. The higher the threshold for multidimensional poverty, the more severe the poverty among UOALA in non-first-tier cities.

**Table 7:** The regional breakdown of the multidimensional poverty index under various values of k, along with the corresponding contribution rates.

k	Multidimensional poverty incidence		Multidimensional poverty index		Contribution rate	
	First-tier city	Non-first-tier city	First-tier city	Non-first-tier city	First-tier city	Non-first-tier city
0.1	0.907	0.951	0.305	0.372	0.367	0.633
0.2	0.729	0.831	0.279	0.354	0.358	0.642
0.3	0.506	0.634	0.225	0.306	0.342	0.658
0.4	0.290	0.456	0.153	0.246	0.305	0.695
0.5	0.160	0.257	0.096	0.157	0.302	0.698
0.6	0.074	0.122	0.050	0.084	0.297	0.703
0.7	0.028	0.049	0.021	0.039	0.277	0.723
0.8	0.006	0.020	0.005	0.017	0.174	0.826
0.9	0.001	0.003	0.001	0.003	0.243	0.757
1.0	0.000	0.000	0.000	0.000	0.000	0.000

### 4.2 Association between family endowments and multidimensional poverty among UOALA

The Dependent variable is whether UOALA are in multidimensional poverty, which is a Binary Dummy Variable. The poverty threshold set by the United Nations Development Program (UNDP) in the Multidimensional Poverty Index is k = 0.33 (32). In this study, we use k = 0.33 as the critical poverty value for UOALA. UOALA who are in multidimensional poverty are assigned a value of1, while those who are not are assigned a value of 0. Half of the UOALA are multidimensionally poor.

#### 4.2.1 Correlation between family endowments and multidimensional poverty among UOALA

With a multidimensional poverty threshold of k = 0.33, the estimation of the correlation between family endowments and multidimensional poverty among UOALA is shown in Table 8. Columns (1)–(3) report the effects of number of children, distance to the nearest child’s residence, and children’s best economic status on whether older adults living alone are multidimensionally poor. The results show that the number of children is not significant, whereas distance to the nearest child’s residence and children’s best economic status both have a significant positive impact at the 1 percent level, with coefficients of 0.120 and 0.281, respectively. This indicates that the closer a child lives and the better their economic status, the higher the probability that an older adult living alone will not be multidimensionally poor. Column (4) includes all three variables simultaneously: the number of children remains insignificant, while distance to the nearest child’s residence and children’s best economic status remain significant at the 1 percent level, with their coefficients rising to 0.149 and 0.305, respectively. The lack of significance in the correlation between the number of children and whether UOALA are in multidimensional poverty suggests that the traditional belief of “more children, more blessings” may no longer be suitable for the current older adults care situation. As the distance between UOALA and children’s residence decreases, intergenerational communication becomes more frequent, providing more assistance to UOALA, leading to better physical and mental health and more active social participation, thus reducing the likelihood of being in multidimensional poverty. This finding is consistent with previous research by Zhou et al. (33), which demonstrated that stronger intergenerational support significantly reduces the risk of multidimensional poverty among urban older adults. Furthermore, a better financial situation for the children provides more economic support to UOALA, improving their living conditions and reducing the likelihood of falling into multidimensional poverty (see Table 9).

**Table 8:** Correlation between family endowments and multidimensional poverty among UOALA.

Variables	k = 0.33				k = 0.2				k = 0.6			
	(1)	(2)	(3)	(4)	(5)	(6)	(7)	(8)	(9)	(10)	(11)	(12)
sum_child	−0.018 (0.048)			0.032 (0.049)	0.061 (0.063)			0.083 (0.064)	0.064 (0.074)			0.104 (0.075)
dis		0.120** (0.056)		0.149*** (0.057)		0.026 (0.066)		0.041 (0.066)		0.065 (0.103)		0.098 (0.107)
inc			0.281*** (0.073)	0.305*** (0.075)			0.141* (0.085)	0.163* (0.086)			0.362*** (0.106)	0.396*** (0.109)
Male	−0.472*** (0.123)	−0.456*** (0.123)	−0.474*** (0.124)	−0.458*** (0.125)	−0.577*** (0.146)	−0.598*** (0.147)	−0.607*** (0.147)	−0.585*** (0.148)	−0.253 (0.204)	−0.259 (0.205)	−0.229 (0.206)	−0.199 (0.206)
Age	0.060*** (0.011)	0.057*** (0.011)	0.059*** (0.011)	0.058*** (0.011)	0.062*** (0.013)	0.067*** (0.013)	0.068*** (0.013)	0.062*** (0.014)	0.065*** (0.019)	0.067*** (0.018)	0.068*** (0.018)	0.059*** (0.020)
hukou	−0.705*** (0.233)	−0.694*** (0.236)	−0.726*** (0.235)	−0.728*** (0.239)	−0.366 (0.316)	−0.373 (0.315)	−0.387 (0.314)	−0.386 (0.317)	−0.595** (0.303)	−0.625** (0.301)	−0.681** (0.304)	−0.682** (0.301)
imm	0.243** (0.108)	0.232** (0.109)	0.226** (0.109)	0.216** (0.110)	0.261** (0.129)	0.254* (0.130)	0.249* (0.130)	0.241* (0.130)	0.235 (0.179)	0.238 (0.181)	0.229 (0.182)	0.210 (0.183)
culture	−0.064 (0.045)	−0.080* (0.045)	−0.035 (0.045)	−0.047 (0.047)	0.042 (0.055)	0.029 (0.055)	0.044 (0.054)	0.052 (0.056)	−0.201** (0.080)	−0.224*** (0.080)	−0.175** (0.078)	−0.167** (0.081)
Mar	−0.178 (0.160)	−0.181 (0.161)	−0.197 (0.162)	−0.191 (0.162)	−0.117 (0.186)	−0.113 (0.186)	−0.114 (0.187)	−0.111 (0.187)	−0.466 (0.287)	−0.487* (0.288)	−0.471* (0.283)	−0.492* (0.285)
City	Control	Control	Control	Control	Control	Control	Control	Control	Control	Control	Control	Control
_cons	−3.938*** (0.890)	−4.085*** (0.895)	−4.713*** (0.911)	−5.067*** (0.937)	−3.752*** (1.074)	−3.997*** (1.077)	−4.406*** (1.085)	−4.330*** (1.099)	−6.677*** (1.606)	−6.765*** (1.582)	−7.691*** (1.604)	−7.650*** (1.637)
*N*	1,601	1,589	1,585	1,582	1,601	1,589	1,585	1,582	1,601	1,589	1,585	1,582
Pseudo R^2^	0.085	0.087	0.092	0.095	0.072	0.072	0.075	0.076	0.081	0.080	0.089	0.092
Chi^2^	149.269	155.563	157.244	168.919	96.837	95.328	97.91	102.936	71.227	72.716	79.466	83.366

The robust standard errors in parentheses *, **, and *** represent significance at the 10, 5, and 1% levels, respectively.

**Table 9:** Gender disparities in the correlation between family endowments and multidimensional poverty among UOALA.

Variables	Male				Female			
	(1)	(2)	(3)	(4)	(5)	(6)	(7)	(8)
sum_child	−0.042 (0.104)			−0.001 (0.107)	−0.015 (0.055)			0.033 (0.057)
Dis		0.110 (0.107)		0.146 (0.110)		0.120* (0.066)		0.145** (0.068)
inc			0.350** (0.145)	0.382*** (0.148)			0.261*** (0.085)	0.280*** (0.087)
Control var.	Control	Control	Control	Control	Control	Control	Control	Control
City	Control	Control	Control	Control	Control	Control	Control	Control
_cons	−4.846*** (1.522)	−4.759*** (1.533)	−5.895*** (1.597)	−6.205*** (1.632)	−3.829*** (1.133)	−4.062*** (1.137)	−4.559*** (1.152)	−4.933*** (1.191)
*N*	461	457	455	454	1,140	1,132	1,130	1,128
Pseudo R^2^	0.128	0.132	0.141	0.143	0.068	0.07	0.074	0.077
Chi^2^	65.033	66.943	69.297	71.847	88.256	92.8	94.31	101.645

The robust standard errors in parentheses *, **, and *** represent significance at the 10, 5, and 1% levels, respectively.

Gender has a significant effect at the 1 percent level: compared to male older adults living alone, female older adults living alone are more likely to fall into a multidimensionally poor state. Age also has a significant effect at the 1 percent level on multidimensional poverty among older adults living alone: as age increases, the probability of being multidimensionally poor rises. UOALA with a rural household registration is more likely to fall into multidimensional poverty than those without, and UOALA who have migrated are more likely to experience multidimensional poverty than those who have not.

To further verify the robustness of the correlation between family endowments and multidimensional poverty among UOALA under different k values, we conducted tests using k = 0.2 and k = 0.6 as the dependent variables for multidimensional poverty among UOALA. The number of children remains not significant, and regardless of whether k is set at 0.2 or 0.6, intergenerational distance becomes no longer significant. This indicates that the correlation between intergenerational distance and multidimensional poverty among UOALA is influenced by the poverty threshold. Neither too low nor too high threshold is correlated with multidimensional poverty among UOALA; significant correlation is only observed at intermediate threshold levels. Children’s income has a relatively robust effect: regardless of the poverty-identification threshold k, t significantly affects multidimensional poverty among older adults living alone. Moreover, as k increases, the level of significance strengthens at k = 0.2, there is a significant positive effect at the 10% level; at k = 0.6, there is a significant positive effect at the 1% level. The larger the k value, the more important children’s income status becomes for determining whether older adults living alone fall into multidimensional poverty. Upon further examination of controlling variables, regardless of the level of poverty threshold, age shows a significant correlation. As age increases, the probability of multidimensional poverty among UOALA also increases. When the poverty threshold is high, gender and migration no longer have a significant correlation, but education level does. When the poverty threshold is low, household registration no longer has a significant correlation.

#### 4.2.2 The correlation between family endowments and multidimensional poverty among UOALA of different genders

Columns (1)–(4) present the estimated effects of family endowment on multidimensional poverty among male older adults living alone. In these specifications, neither number of children nor residential distance has a significant effect on multidimensional poverty for male older adults. However, children’s income has a significant positive effect at the 1 percent level (coefficient = 0.382), indicating that the better the children’s economic status, the lower the probability that a male older adult living alone is multidimensionally poor.

Columns (5)–(8) report the estimated effects of family endowment on multidimensional poverty among female older adults living alone. Here, number of children remains insignificant, while children’s residential distance and income each have a significant positive effect—at the 5 percent level for distance (coefficient = 0.145) and at the 1 percent level for income (coefficient = 0.280). This implies that, for female older adults living alone, closer intergenerational proximity and better children’s economic status both reduce the probability of falling into multidimensional poverty.

#### 4.2.3 Regional disparities in the correlation between family endowments and multidimensional poverty among UOALA

The regional disparities in family endowments on multidimensional poverty among UOALA reveal that the estimates in columns (1) to (4) depict the correlation between first-tier city family endowments and multidimensional poverty among UOALA, while columns (5) to (8) showcase the correlation between non-first-tier city family endowments and multidimensional poverty among UOALA (see Table 10). Regardless of whether it is a first-tier city or non-first-tier city, the influence of the number of children on multidimensional poverty among UOALA is not significant. Children’s income status remains robust, exhibiting a significant positive effect at the 1 percent level in both first-tier and non-first-tier cities, with coefficients of 0.335 and 0.247, respectively. Distance to the nearest child’s residence has no significant effect for older adults living alone in first-tier cities, but for those in non-first-tier cities it has a significant positive effect at the 1 percent level (coefficient = 0.247). This may be attributed to the fact that compared to non-first-tier cities, first-tier cities have a more comprehensive social security and service system, reducing the UOALA’s dependence on close care from their children. In contrast, non-first-tier cities, constrained by the level of older adults care services, rely more on informal care from children’s families.

**Table 10:** Regional disparities in the correlation between family endowments and multidimensional poverty among UOALA.

Variables	First-tier city				Non-first-tier city			
	(1)	(2)	(3)	(4)	(5)	(6)	(7)	(8)
sum_child	−0.024 (0.072)			0.018 (0.074)	0.036 (0.062)			0.098 (0.064)
Dis		0.041 (0.080)		0.054 (0.080)		0.278*** (0.076)		0.327*** (0.077)
inc			0.335*** (0.115)	0.335*** (0.117)			0.188** (0.087)	0.247*** (0.090)
Control	Control	Control	Control	Control	Control	Control	Control	Control
_cons	−7.509*** (1.280)	−7.513*** (1.284)	−8.299*** (1.313)	−8.356*** (1.343)	2.312** (1.160)	1.757 (1.184)	1.700 (1.200)	0.884 (1.232)
*N*	673	672	672	671	928	917	913	911
Pseudo R^2^	0.051	0.051	0.06	0.06	0.019	0.031	0.022	0.039
Chi^2^	44.876	44.355	51.066	50.546	23.711	33.886	26.812	43.576

The robust standard errors in parentheses. **, and *** represent significance at the 5%, and 1% levels, respectively.

#### 4.2.4 Gender disparities in the correlation between family endowments and multidimensional poverty among UOALA

Based on the estimates of the correlation between family endowments and multidimensional poverty among UOALA by gender in Table 11, columns (1) to (4) present the estimates of the correlation between son characteristics and multidimensional poverty among UOALA, while columns (5) to (8) present the estimates of the correlation between daughter characteristics and multidimensional poverty among UOALA. The results show that, for son-specific characteristics, only sons’ income status has a significant positive effect on multidimensional poverty among older adults living alone at the 1 percent level (coefficient = 0.336), while the number of sons and residential distance are not significant. For daughter-specific characteristics, daughters’ residential distance and income both have significant positive effects at the 5 percent level (coefficients = 0.137 and 0.229, respectively). However, when number of daughters, residential distance, and income are all included in the model simultaneously, daughters’ residential distance is no longer significant, and only daughters’ income remains significant at the 5 percent level (coefficient = 0.244).

**Table 11:** Estimates of gender disparities in the correlation between family endowments and multidimensional poverty among UOALA.

Variables	Son				Daughter			
	(1)	(2)	(3)	(4)	(5)	(6)	(7)	(8)
sum_son	−0.048 (0.055)			−0.005 (0.086)				
dis_son		0.100 (0.061)		0.087 (0.077)				
inc_son			0.316*** (0.100)	0.336*** (0.100)				
sum_dau					0.020 (0.052)			−0.034 (0.088)
dis_dau						0.137** (0.062)		0.119 (0.083)
inc_dau							0.229** (0.109)	0.244** (0.112)
Control	Control	Control	Control	Control	Control	Control	Control	Control
City	Control	Control	Control	Control	Control	Control	Control	Control
_cons	−3.974*** (0.886)	−4.147*** (1.004)	−5.317*** (1.245)	−5.679*** (1.273)	−3.863*** (0.881)	−4.287*** (1.001)	−4.014*** (1.347)	−4.292*** (1.381)
Pseudo R^2^	0.085	0.091	0.097	0.098	0.085	0.089	0.093	0.095
Chi^2^	149.483	131.26	93.047	95.718	149.576	122.034	69.833	73.53

The robust standard errors in parentheses. **, and *** represent significance at the 5%, and 1% levels, respectively.

## 5 Conclusion

The study examined the state of multidimensional poverty among UOALA in China using the AF method. The results indicate that income is not the sole cause of poverty among UOALA. From a multidimensional perspective, the scale and depth of poverty among UOALA have expanded. Approximately 80% of the sampled UOALA are in poverty in at least one dimension, with nearly one-tenth of them experiencing poverty in at least three dimensions. It is evident that the issue of multidimensional poverty cannot be overlooked within the group of UOALA.

The decomposition of deprivation indicators in each dimension (Table 3) reveals that the contribution rates of each dimension to overall poverty among UOALA vary. Impoverished UOALA suffer more severe deprivation in terms of physical and mental health. When examining specific subgroups, the contribution rates of indicators also differ. There exists significant subgroup inequality in the multidimensional poverty among UOALA, with vulnerable groups such as females, and those living outside first-tier cities experiencing higher poverty incidence and depth. This highlights the critical importance of tailored poverty alleviation policies that are responsive to local conditions and individual needs. In addressing the various factors driving into poverty, various targeted poverty reduction measures should be implemented in response to specific needs or challenges, with a focus on building targeted safeguard systems for different subjects, levels, and categories.

Furthermore, this study also explored the intricate relationship between family endowments and multidimensional poverty among UOALA. The family serves as the fundamental domain for the later years of older adults, with the genesis of poverty in old age intricately linked to the family unit. Previous research has often focused on the household unit as a whole, using cohabitation or household registration as the basis of family structure analysis. However, this approach may obscure the poverty experienced by certain UOALA and the underlying root causes of some poverty issues. Chinese families are characterized by strong practical relationships, and through a survey conducted using subjective family concepts, it was found that the number of children does not significantly correlate with multidimensional poverty among UOALA. Conversely, factors such as the proximity of children’s residences and their financial situations are significantly related to the occurrence of multidimensional poverty among UOALA. In terms of regional disparities, children’s economic status is a common correlating factor, while intergenerational residential distance is particularly significant in non-first-tier cities in relation to multidimensional poverty among UOALA. When considering the gender of children, it was observed that only the income of sons correlates with multidimensional poverty among UOALA, whereas daughters exhibit correlations in both income and residential distance.

With deepening population aging and accelerated social transformation, the phenomenon of older adults living alone has become increasingly common, especially among the very old. Compared to other living arrangements, older adults living alone not only face higher living costs and greater economic pressure but also encounter multidimensional poverty risks—spanning health, social participation, and functional ability, among others. However, current old-age assistance policies and systems largely overlook how the proportion of older adults in a household, household structure, and living arrangements affect family members’ welfare, particularly that of older adults (6). Therefore, when implementing assistance measures, local authorities should prioritize older adults living alone, approaching their needs from a multidimensional poverty perspective and offering comprehensive support. The benefit standards should be raised to ensure that older adults living alone have sufficient financial resources to meet basic needs; special subsidies—such as medical subsidies or daily care allowances—should be established to address their unique requirements; the diversified activities suitable for the older adults should be carried out to increase their social participation and enrich their spiritual life.

In China, the family is a relationship network that transcends time and space. Although the social old-age care system is gradually developing, in the short term, a family’s resource endowment continues to play a crucial role in parents’ care in old age (8). Therefore, policy design should extend beyond focusing solely on individual welfare for older adults to include attention to their intergenerational families, recognizing the social value of family caregivers and attempting to implement support policies that treat the family as a whole. In this way, “home” can truly become a haven for older adults living alone to enjoy their later years. Simultaneously, when formulating policy, the government must design more humane housing policies to shorten the spatial distance between older adults and their children. By implementing diverse measures—such as public subsidies, caregiver training, and online medical consultations—it can effectively enhance children’s capacity and resources for caring for older adults, thereby providing more solid support for family-based care.

The multidimensional perspective on poverty measurement provides a more valuable theoretical and empirical reference for understanding and targeting the impoverished population among UOALA. Although standardized multidimensional poverty measurement indicators have not yet been established in China, scholars are exploring more practical multidimensional poverty standards. In the future, replacing the traditional economic poverty line with multidimensional poverty will emerge as a new trend in poverty identification, targeted poverty alleviation, and prevention of relapse into poverty in China. However, certain limitations remain. Because this study uses cross-sectional data from a single year, it lacks comparative research across time periods and regions and does not include dynamic analyses of national policy implementation or social structural changes; consequently, it cannot reveal cohort-period effects. Moreover, this study’s sample is limited to older adults living alone in urban areas, and it does not examine existing urban–rural differences. Given China’s pronounced urban–rural dual structure, research on regional heterogeneity would help identify more generalizable patterns. Future studies should pay greater attention to these issues and explore them in depth.

## Data Availability

Publicly available datasets were analyzed in this study. This data can be found at: https://fz.people.com.cn/skygb/sk/index.php/index/seach?pznum=12%26ZD212&xmtype=0&xktype=0&xmname=&lxtime=0&xmleader=&zyzw=0&gzdw=&dwtype=0&szdq=0&ssxt=0&cgname=&cgxs=0&cglevel=0&jxdata=0&jxnum=&cbs=&cbdate=0&zz=&hj=250630.
